# Impact of cardiac rehabilitation on erectile dysfunction in cardiovascular patients: a systematic review and meta-analysis

**DOI:** 10.1093/sexmed/qfae043

**Published:** 2024-07-01

**Authors:** Masoumeh Sadeghi, Ali Askari, Fatemeh Bostan, Afshin Heidari, Hamed Rafiee, Ghazaal Alavi Tabatabaei, Golsa Ghasemi, Hamidreza Roohafza

**Affiliations:** Chamran Cardiovascular Research and Education Hospital, Isfahan University of Medical Sciences, Isfahan, Iran; Cardiac Rehabilitation Research Center, Cardiovascular Research Institute, Isfahan University of Medical Sciences, Isfahan, Iran; Cardiac Rehabilitation Research Center, Cardiovascular Research Institute, Isfahan University of Medical Sciences, Isfahan, Iran; School of Medicine, Isfahan University of Medical Sciences, Isfahan, Iran; Cardiac Rehabilitation Research Center, Cardiovascular Research Institute, Isfahan University of Medical Sciences, Isfahan, Iran; School of Medicine, Isfahan University of Medical Sciences, Isfahan, Iran; Cardiac Rehabilitation Research Center, Cardiovascular Research Institute, Isfahan University of Medical Sciences, Isfahan, Iran; School of Medicine, Isfahan University of Medical Sciences, Isfahan, Iran; Health Policy Research Center, Shiraz University of Medical Sciences, Shiraz, Iran; School of Medicine, Isfahan University of Medical Sciences, Isfahan, Iran; School of Medicine, Isfahan University of Medical Sciences, Isfahan, Iran; School of Medicine, Isfahan University of Medical Sciences, Isfahan, Iran; Isfahan Kidney Diseases Research Center, Isfahan University of Medical Sciences, Isfahan, Iran; Isfahan Cardiovascular Research Center, Cardiovascular Research Institute, Isfahan University of Medical Sciences, Isfahan, Iran

**Keywords:** cardiac rehabilitation, erectile dysfunction, cardiovascular diseases, quality of life, International Index of Erectile Function, IIEF

## Abstract

**Background:**

Cardiovascular diseases (CVDs) and erectile dysfunction (ED) frequently co-occur, significantly affecting the quality of life of individuals.

**Aim:**

To assess the impact of cardiac rehabilitation (CR) on ED in patients with CVD through a systematic review and meta-analysis.

**Methods:**

This study analyzed randomized controlled trials and other studies comparing CR with usual care for adult males (≥18 years) with any cardiac disease. Literature searches were extensive, and the risk of bias was evaluated by the Cochrane Collaboration tool. Data from 6 studies involving 668 participants were included in the meta-analysis.

**Outcomes:**

The primary outcome was the improvement in ED, as measured with the International Index of Erectile Function.

**Results:**

A statistically significant improvement in erectile function was observed across 6 studies, with a Morris dppc2 effect size of 0.38 (95% CI, 0.17-0.59). Despite initial high heterogeneity (*I*^2^ = 95.7%), identification and correction for selective outcome reporting bias mitigated this issue.

**Clinical Translation:**

CR has a modest but statistically significant impact on improving ED in patients with CVD, indicating its potential positive contribution to the quality of life of this group.

**Strengths and Limitations:**

The study’s strengths include a comprehensive literature search and a rigorous methodological approach. Limitations involve high heterogeneity among studies and a low level of evidence due to small sample sizes and study quality; however, the source of heterogeneity was identified and mitigated following risk-of-bias assessment.

**Conclusion:**

The results suggest that CR has a statistically significant but modest impact on improving ED in patients with CVD. Clinicians should consider the integration of CR into the clinical management of these individuals. This study underscores the potential for CR to contribute positively to the quality of life for patients with CVD by addressing associated ED (PROSPERO: CRD42022374625).

## Graphical Abstract

**Graphical Abstract:**
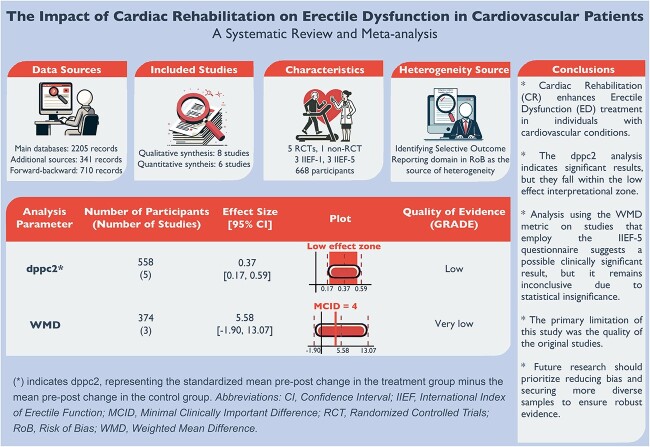

## Introduction

Erectile dysfunction (ED), formerly known as impotence (now considered an obsolete term),^1^ is characterized by the difficulty of attaining and retaining the penile erection, leading to suboptimal sexual satisfaction. This clinical condition can result in reduced quality of life (QoL) and compromised self-esteem.^2^ Of the estimated 150 million men worldwide with ED, this condition is more prevalent among the older segment of the male population, with the incidence of impotence rising with age.^3^ In addition to aging, cardiovascular diseases (CVDs) can contribute to ED, with a 42%-57% prevalence of ED in patients with CVD.^3^^,^^4^ These conditions are companions since they share similar risk factors, such as hypertension, obesity, and smoking.^3^ Additionally, endothelial dysfunction and atherosclerosis can affect the penis and heart and lead to ED as well as CVD.^5^ It is also notable that some CVD medications (thiazides, calcium channel blockers, and angiotensin-converting enzyme inhibitors) can interfere with proper erectile function.^6^ CVDs are particularly noteworthy as they lead to the presumption that every patient with CVD should be rigorously evaluated for potential ED. Similarly, this suggests that each patient with ED may have an underlying cardiac or vascular issue, even in the absence of symptoms, until proven otherwise.^3^ Moreover, ED is an excellent barometer of overall cardiovascular health. Research has shown that it typically precedes a cardiovascular event by an average of 2 to 5 years. Men with ED should be evaluated for underlying cardiac disease until proven otherwise, following the Princeton III consensus recommendations.^7^^,^^8^

Given the impact of CVDs on overall health and erectile function, coupled with the effect of ED on QoL, it is imperative to develop strategies to improve erectile function. Cardiac rehabilitation (CR) is described as a productive and beneficial secondary prevention program rooted in physical activity.^9^ CR primarily comprises multiple components, such as baseline health status assessment, dietary guidance, psychoeducational counseling, risk factor modification, and exercise consultation and education.^10^ Propitiously, CR offers multiple benefits, including enhancing psychological well-being and QoL, decreasing the mortality rate associated with CVDs, and promoting overall health.^11^ Given that ED is a companion with CVDs,^12^ it is anticipated that CR may be effective in augmenting erectile function with pharmacotherapy. The reason is that CR addresses psychosocial disorders, fosters a relationship between patients and caregivers, and facilitates regular physical activities for the patient.^13^

CR has shown some promising results in improving various aspects of sexual health in patients with CVD. Studies have found that participation in CR programs is associated with improvements in ED, sexual activity, and sexual satisfaction.^14-16^ The multifaceted nature of CR, which includes exercise training, risk factor modification, and psychosocial support, is thought to positively influence sexual function through physiologic and psychological mechanisms.^17^^,^^18^ However, the specific impact of CR on sexual dysfunction in patients with CVD warrants further investigation.

Upon reviewing the original research articles on this topic, we identified inconsistent and varying outcomes across the literature.^19-21^ Accordingly, to obtain a comprehensive understanding of the overall effect and identify potential sources of heterogeneity, conducting a meta-analysis was essential. Furthermore, the primary studies were limited in power, mainly due to being trials with small sample sizes.^15^^,^^16^^,^^19-24^ This underscored the need for a meta-analysis with enhanced statistical power to derive conclusive results. Upon evaluation of previously published systematic reviews, none fully addressed the posed question.^25-28^ However, 1 study exhibited similarities to our topic.^29^ Despite the thematic overlap with this study, we identified compelling reasons to undertake our systematic review and meta-analysis. The prior study was constrained by a limited range of databases in its search strategy and reported data exclusively with the standardized mean difference effect size. In contrast, our research utilized weighted mean difference (WMD) and Morris^30^ dppc2, as well as the GRADE checklist tool (Grading of Recommendations Assessment, Development and Evaluation)^31^ to assess the evidence’s strength.

Given our understanding of the pivotal role that CR plays in ameliorating ED among patients with CVD, this systematic review and meta-analysis aims to rigorously assess the impact of CR on ED in individuals with established CVD. This study aims to test the hypothesis that CR improves ED in patients with CVD.

## Methods

### Study design

To ensure the clarity of this study, the protocol for this systematic review and meta-analysis was registered with the International Prospective Register of Systematic Reviews (PROSPERO: CRD42022374625). This review was accomplished scrupulously and written under the adjusted framework of the PRISMA guidelines (Preferred Reporting Items for Systematic Reviews and Meta-analyses).^32^ Additionally, we followed PRISMA extensions—namely, PRISMA 2020 for abstracts^33^ and the PRISMA-S checklist^34^ (Supplementary Methods 1-3).

### Search strategy

To undertake a systematic literature search, several electronic bibliographic databases, including MEDLINE (via PubMed), Scopus, Embase, Web of Science, and CENTRAL (Cochrane Central Register of Controlled Trials), were explored on November 30, 2022, by 1 of our authors (A.A.) for English-language randomized controlled trials (RCTs) and non-RCTs from January 2000 to October 2022. In addition to these 5 primary databases, we searched clinical trial protocols on ClinicalTrials.gov, the International Clinical Trials Registry Platform, and the International Standard Randomized Controlled Trial Number as secondary sources to identify relevant registered clinical trials. Our search strategy incorporated Medical Subject Headings terms and the 3-phase method^35^ to pinpoint synonyms for elements of 2 core domains: (1) CR, aligning with the intervention component of the PICO framework (population, intervention, control, and outcomes), which included terms synonymous with “cardiovascular rehabilitation” and “cardiac rehabilitation”; (2) phrases such as “erectile function,” “erectile dysfunction,” “male impotence,” “male sexual impotence,” “impotence,” and others relevant to the study’s primary outcome, which focused on changes in erectile function in patients with CVD following CR. The detailed search syntax is available in Supplementary Method 4.

Ultimately, to access the gray literature, OpenGrey, ProQuest, and Open Access Theses and Dissertations were searched, along with the key journal. Furthermore, we employed the forward-and-backward citation search methodology^36^ to explore references and studies that cited our primary articles.

### Study selection and eligibility criteria

Following the comprehensive search, identified articles were imported into Mendeley Desktop reference management software (version 1.19.8; Mendeley Ltd), where duplicate entries were systematically removed. During the initial screening phase, studies were preliminarily included by title and abstract following independent assessment by 2 authors (H.R. and G.G.). Afterward, in the selection phase, 2 other authors (A.A. and G.G.) independently evaluated and chose the primary studies based on the potential eligibility of their full texts. Disagreements were first resolved through discussion between the authors to reach a consensus. A third researcher (A.A.) was responsible for facilitating a resolution if the consensus was not achieved during the screening phase.

Selection decisions were based on the study design, characteristics of the intervention group, and eligibility criteria. In terms of research design, this review focused on interventional studies, specifically RCTs and non-RCTs, excluding descriptive, comparative, and qualitative studies. For the intervention group, the target was any type of CR program encompassing exercise-based programs and/or psychosocial consultations. Eligibility was limited to adult males (aged ≥18 years) diagnosed with any cardiac disease or those who had undergone cardiovascular surgery, such as coronary artery bypass graft. Conversely, studies targeting adolescents aged <18 years or females were excluded.

This article, designed as a systematic review and meta-analysis, did not require institutional review board approval, as it involves the analysis of previously published articles. However, we ensured that all studies had obtained ethical approvals from their institutions and documented informed consent from participants.

### Data extraction

In recognition of the precision required in data extraction, quantitative data were fastidiously collected by 2 authors (F.B. and G.A.T.) utilizing distinct preconstructed Google Sheets, a free online spreadsheet tool (Google LLC). After consensus between the authors, data were consolidated into a final sheet. In instances of discrepancies, another researcher (A.A.) cross-referenced the primary extraction forms with the full text of the articles. The data extraction sheets captured (1) bibliographic details and study characteristics, including DOI, first author, study design, country, publication year, intervention duration, follow-up instances, and erectile function assessment tools; (2) demographic attributes such as age, smoking status, marital status, and comorbidities; (3) details on CR and its components; and (4) the study’s outcome measure, particularly assessing potential changes in erectile function post-CR with the International Index of Erectile Function (IIEF) or other validated questionnaires.

In cases where information was absent, weekly emails were dispatched to the corresponding author of the relevant article over a span of 3 weeks. In the instance of receiving no reply, the study was promptly excluded if the missing data pertained to the primary outcome.

### Risk-of-bias assessment

To evaluate the quality of the primary studies, 2 authors (H.R. and G.G.) independently employed the Cochrane Collaboration tool for assessing the risk of bias (RoB).^37^ Various domains of this tool were utilized for the RoB assessment, including sequence generation, allocation concealment, blinding of participants, incomplete outcome data, selective outcome reporting, and other sources of bias. The sixth domain was tailored to assess baseline imbalance, gauging the study’s randomization quality and efficacy. Furthermore, allocation concealment and incomplete data were omitted due to minimal variance among studies. Ultimately, each study was categorized as high quality if there was a low RoB in 3 or 4 domains and low quality if <3 domains showed a low RoB.

All discrepancies were addressed either by reaching a consensus between the authors or by seeking the opinion of a third person if an agreement was not achieved.

### Quality of evidence

To assess the strength and quality of evidence, the study outcome (changes in erectile function) was evaluated per the modified version of the GRADE checklist tool^31^ by 1 of the authors (A.A.). The baseline score was set at 4, as RCTs and non-RCTs were included in this study. Additionally, only the downgrading elements of this tool were applied (RoB, inconsistency, imprecision, and publication bias), excluding the upgrading factors; hence, the maximum score achievable remained 4 (score ≤4). For evidence quality, scores were interpreted as 4 for high quality, 3 for moderate, 2 for low, and 1 or below for very low.

### Statistical analysis

Statistical analyses were performed with Stata software (version 14; StataCorp LLC) to evaluate the impact of CR on ED in cardiovascular patients. Given the substantial methodological heterogeneity, such as study design, patient demographics, CR components, and assessment tools observed among the primary studies, a random effects model was deemed appropriate for our analysis. Findings are presented via forest plots, which include 95% CIs. *P* < .05 was established to determine statistical significance.

Effect sizes were calculated per the Morris dppc2 formula,^30^ representing the standardized mean difference, where each unit is equivalent to 1 SD. The effect sizes were categorized as negligible (0≤0.2), small (0.2-0.49), moderate (0.5-0.79), or large (≥0.8).^38^ Additionally, WMD was used for studies utilizing the 5-item IIEF (IIEF-5).

Subgroup analyses were conducted to explore potential sources of heterogeneity among the studies. To assess publication bias, initial analyses were performed with Begg’s and Egger’s tests, followed by a trim-and-fill analysis for further evaluation. For assessing publication bias, *P* < .1 was considered indicative of statistical significance. Finally, a sensitivity analysis employing the 1-study-removed approach was conducted to assess the robustness of our results.

## Results

### Study selection and characteristics

**Figure 1:**
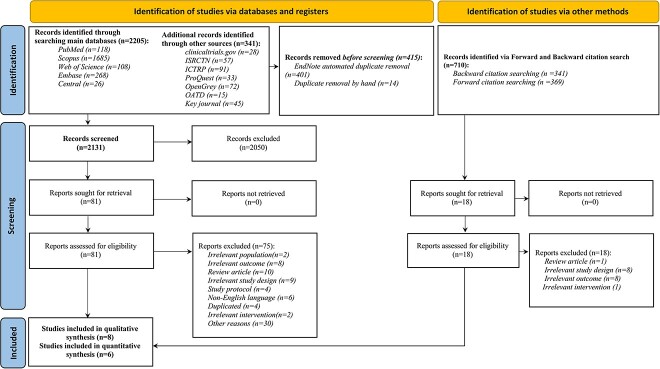
PRISMA 2020 flow diagram for the systematic review stages: the PRISMA flow diagram details the systematic review methodology, illustrating the phases of study identification, screening, and inclusion. CENTRAL, Cochrane Central Register of Controlled Trials; ICTRP, International Clinical Trials Registry Platform; IIEF, International Index of Erectile Function; ISRCTN, International Standard Randomized Controlled Trial Number; OATD, Open Access Theses and Dissertations; PRISMA, Preferred Reporting Items for Systematic Reviews and Meta-analyses.

From a thorough search across primary databases, registers, and other sources, 2546 articles were initially identified. After accounting for duplicates and applying a rigorous 2-phase screening process complemented by a forward-backward citation search, 8 studies were found suitable for inclusion in the systematic review. Among these 8 studies, 6 were incorporated into the meta-analysis.^15^^,^^19-21^^,^^23^^,^^24^ One study was excluded due to its categorical outcome reporting,^16^ and another was omitted as it employed a bespoke questionnaire for ED assessment not aligned with the standardized measures used in the other studies^22^ (Figure 1).

The meta-analysis involved 668 participants across 6 distinct studies. The investigations utilized distinct study designs: 5 studies were RCTs, and 1 was a non-RCT cohort study. Geographic dispersion was notable, with 4 conducted in Asian countries and 2 in European countries. The clinical cardiovascular conditions observed among the studies’ participants were diverse: 2 assessed patients with a history of coronary artery bypass graft; 2 examined patients with ischemic heart disease; 1 defined the population as patients with coronary heart disease; and 1 studied individuals with coronary artery disease.

Regarding the evaluation metrics for ED, 3 investigations implemented the IIEF-5, while the other 3 utilized the IIEF. Other characteristics, such as control and intervention group modalities, total number of sessions, and session frequency and duration, are summarized in Table 1.

**Table 1:** Characteristics of studies in the systematic review and meta-analysis.

						**Assessment**		**Sessions**			**Group care**	
**Study**	**Design**	**Country**	**CV Diagnosis**	**No.**	**Age, y, mean ± SD**	**Tool**	**Baseline, mean ± SD**	**No.**	**Times/ wk**	**Duration, min**	**Intervention**	**Control**
Kaikhosro Doulatyari (2019)^21^^,^^a^	RCT	Iran	CABG	126	59.6 (8)	IIEF-5	14.4 (4.9)^b^	>26, both groups; >60 Kegel	3	90	Cardiac rehabilitation, Kegel, psychoeducation	Cardiac rehabilitation
Palm (2019)^19^^,^^a^	RCT	Denmark	IHD	154	61.6 (6.1)	IIEF	9.8 (9.8)	36	3	60	Physical exercise, pelvic floor exercise, psychoeducation, PDE5i if needed	Usual care, PDE5i if needed
Pournaghash-Tehrani (2014)^20^^,^^a^	RCT	Iran	CABG	110	59.3 (7.1)	IIEF-5	9.4 (2.3)	8	—	—	PRECEDE-PROCEED model	Regular rehabilitation training, nutrition and psychological counseling
Kałka (2013)^15^^,^^a^	RCT	Poland	IHD	138	62.1 (8.6)	IIEF-5	12.4 (5.9)^b^	30	5	45	Cardiac rehabilitation, pharmacotherapy	Pharmacotherapy
Rehana (2022)^24^^,^^a^	Non-RCT	Indonesia	CHD	60	60.5 (3.6)	IIEF	16.1 (3.7)^b^	18	3	35	Education, walking, cycling, jogging	Based on doctor’s recommendation
Tirgari (2019)^23^^,^^a^	RCT	Iran	CAD	80	52.9 (9.3)	IIEF	9.8 (4.4)	5	1	20	Exercise, education	Usual care
Begot (2015)^16^	RCT	Brazil	AMI and successful PCI	86	57.9 (9.4)	IIEF	—	12	4	50	Home-based walking, education	Usual care, education
Belardinelli (2005)^22^	RCT	Italy	Stable CHF	59	56.9 (13.5)	SAPQ	—	24	3	60	Exercise	Usual care

Abbreviations: AMI, acute myocardial infarction; CABG, coronary artery bypass grafting; CAD, coronary artery disease; CHD, coronary heart disease; CV, cardiovascular; IHD, ischemic heart disease; IIEF, International Index of Erectile Function; IIEF-5, 5-item International Index of Erectile Function; PCI, percutaneous coronary intervention; PDE5i, phosphodiesterase type 5 inhibitor; RCT, randomized controlled trial.

a Studies in the meta-analysis.

b Data adjusted to a scale of 25.

### Efficacy of CR on ED

The effectiveness of CR programs on ED was evaluated by Morris dppc2, revealing a pooled effect size of 1.08 (95% CI, 0.09-2.06). Notable heterogeneity among the studies was observed, with a high *I*^2^ value of 95.7% (*P* < .001; Figure S1).

**Figure 2:**
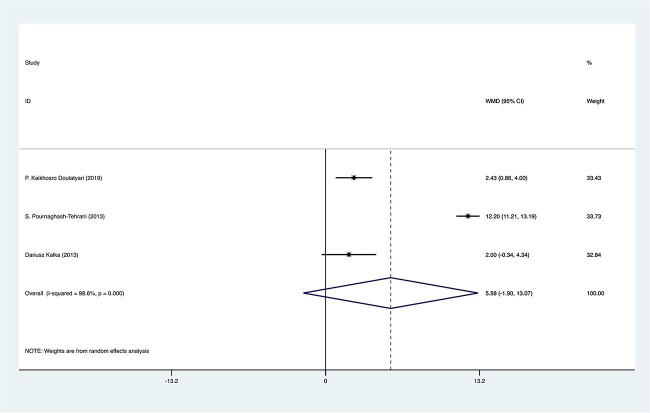
Weighted mean difference (WMD)–based forest plot assessing cardiac rehabilitation effects on erectile dysfunction in studies employing the IIEF-5: this forest plot summarizes the cardiac rehabilitation effect on erectile dysfunction, quantified by WMD in the studies that utilized the IIEF-5. The WMD and 95% CIs were calculated via a random effects meta-analysis. Heterogeneity among studies was assessed with the *I*^2^ statistic and Cochran’s *Q* test. ES, effect size; IIEF-5, 5-item International Index of Erectile Function.

In the studies that utilized the IIEF-5 as their evaluation tool, the calculated WMD was 5.58 (95% CI, –1.90 to 13.07). A high level of heterogeneity among these studies was also evident, as indicated by an *I*^2^ value of 98.6% (*P* < .001; Figure 2).

Two additional studies not in the meta-analysis showed comparable results. The Begot et al^16^ study was excluded from the meta-analysis due to categorical outcome reporting, and the Belardinelli et al^22^ study was excluded due to the use of a bespoke questionnaire not aligned with standardized measures, particularly the IIEF, used by other studies in the meta-analysis. The study by Begot et al observed that ED went up by 9% in the control group (*P* = .08) but decreased by 71% in the intervention group doing walking exercises (*P* < .0001). Belardinelli et al also saw a major pre- to posttest improvement in sexual activity scores, going from an initial average of 3.49 to 6.17 at the end (*P* < .001).

### Risk of bias

**Figure 3:**
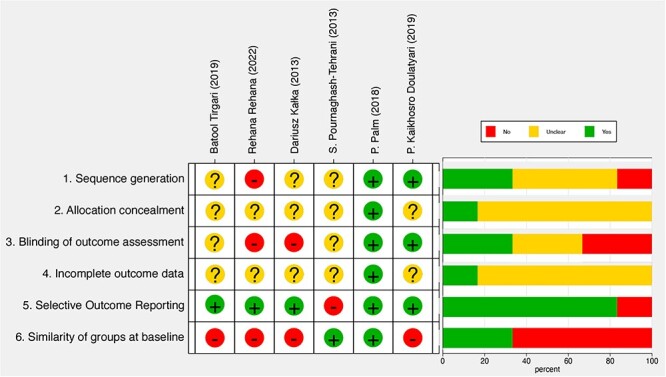
Traffic light and summary plot of risk of bias (RoB): this figure presents a comprehensive visual representation of the RoB assessment conducted per the Cochrane Collaboration tool. This summary plot employs a traffic light color-coding system to depict the level of RoB in various studies. Indicators represent studies deemed to be free of RoB within a specific domain, those with RoB, and instances where the RoB remains unclear or undetermined.

The study of Palm et al^19^ consistently displayed a low RoB across all areas and will not be discussed further. In contrast, other studies showed mixed levels of bias in specific areas. For sequence generation (domain 1), Kaikhosro Doulatyari et al^21^ had low RoB, Rehana et al^24^ had high RoB, and 3 others had some concerns. For allocation concealment (domain 2) and incomplete outcome data (domain 4), all 5 studies showed some bias. For blinding of participants (domain 3), Kaikhosro Doulatyari et al was low in bias, Kałka et al^15^ and Rehana et al were high, and 2 others raised concerns. In selective outcome reporting (domain 5), all except Pournaghash-Tehrani et al^20^ had low bias. For the similarity of groups at baseline (domain 6), all but Pournaghash-Tehrani et al showed high RoB. Details are provided in Figure 3.

### Subgroup analyses

Table 2 presents the subgroup analysis outcomes, with effect sizes reported as dppc2. Out of all conducted subgroup examinations, 5 manifested statistically significant intergroup variability.

**Table 2:** Subgroup analysis assessing the impact of study characteristics on heterogeneity.^a^

			**Within-subgroup heterogeneity**		**Between-subgroup heterogeneity**
**Potential factor: subgroup**	**dppc2 (95% CI)**	**No. of studies**	***I*** ^**2**^ **, %**	***P* value**	***P* value**
Design					.159
RCT	1.24 (0.08, 2.40)	5	96.5	**<.001**	
Non-RCT	1.08 (0.09, 2.06)	1	—	—	
Country					**.001**
Developing	1.50 (–0.15, 3.15)	4	97.1	**<.001**	
Developed	0.29 (–0.01, 0.59)	2	0.0	.943	
Tool					**.008**
IIEF-5	1.76 (–0.43, 3.95)	3	98.1	**<.001**	
IIEF	0.41 (0.13, 0.70)	3	0.0	.403	
Overall quality					**.002**
High	0.35 (0.06, 0.64)	2	0.0	.719	
Low	1.47 (–0.23, 3.17)	4	97.2	**<.001**	
Without selective outcome reporting bias					**<.001**
Yes	0.38 (0.17, 0.59)	5	0.0	.722	
No	4.68 (3.92, 5.44)	1	—	—	
No. of sessions					**<.001**
≥27	0.33 (0.09, 0.57)	3	0.0	.905	
<27	1.88 (–0.56, 4.32)	3	97.8	**<.001**	
Session duration					.754
≥1 h	0.35 (0.06, 0.64)	2	0.0	.719	
<1 h	0.42 (0.12, 0.72)	3	0.0	.397	
Overall	1.08 (0.09, 2.06)	6	95.7	**<.001**	NA

Abbreviation: NA, not applicable.

a This table presents a subgroup analysis examining various health factors, intervention dosages, and study quality while detailing dppc2 with a 95% CI, calculated via a random effects meta-analysis. Bold values indicate *P* < .05, denoting statistical significance from between-subgroup heterogeneity tests. Within the subgroup, heterogeneity was assessed with the *I*^2^ test and Cochran’s *Q* test.

Studies from developing countries showcased a pronounced effect size as compared with their counterparts in developed nations (1.50 [95% CI, –0.15 to 3.15] vs 0.29 [95% CI, –0.01 to 0.59]). When ED measurement tools were compared, studies employing the IIEF-5 yielded more substantial effect sizes (1.76; 95% CI, –0.43 to 3.95) than those using the IIEF (0.41; 95% CI, 0.13-0.70). A divergence was also noted in studies based on the number of intervention sessions, with those having fewer sessions demonstrating a larger effect size than those with extended sessions (1.88 [95% CI, –0.56 to 4.32] vs 0.33 [95% CI, 0.09-0.57]).

### Quality analysis

**Figure 4:**
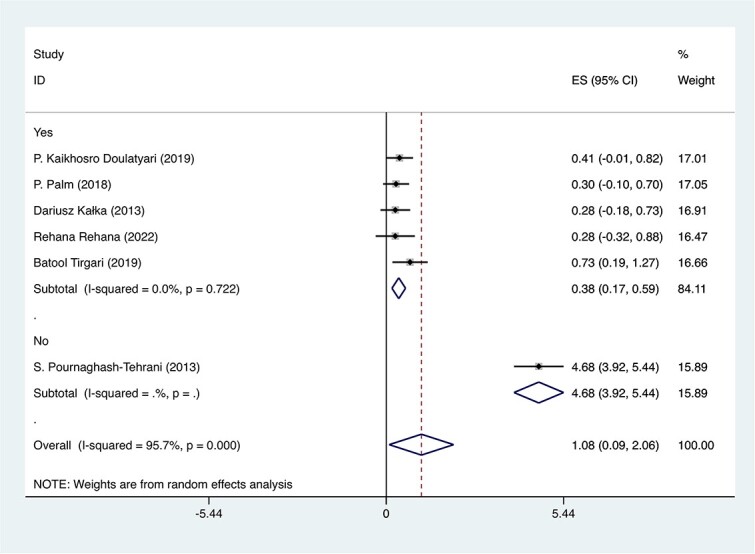
dppc2 forest plot stratified by selective outcome reporting in risk of bias: this forest plot displays the stratification of studies by the selective outcome reporting domain of the risk-of-bias assessment, utilizing the dppc2 metric. The dppc2 and 95% CIs were calculated via a random effects meta-analysis. Heterogeneity among studies was assessed with the *I*^2^ statistic and Cochran’s *Q* test. ES, effect size.

Assessments stratified by RoB revealed that studies categorized as lower quality (1.47; 95% CI, –0.23 to 3.17) had a more significant effect size than those marked with low RoB (0.35; 95% CI, 0.06-0.64; Figure S2). The substantial difference underscores a pronounced association between study qualities and their findings. Excluding Palm et al,^19^ which consistently displayed low RoB across domains, and with the exception of Pournaghash-Tehrani et al,^20^ all studies indicated a low RoB in the fourth domain, selective outcome reporting. This domain-based subgroup analysis elucidated that studies with low RoB had a diminished effect size relative to Pournaghash-Tehrani et al (0.38 [95% CI, 0.17-0.59] vs 4.68 [95% CI, 3.92-5.44]; Figure 4). Additionally, this pattern of studies with low RoB demonstrating a higher effect size when compared with those with higher RoB was consistent across all other domains, as depicted in Figures S3 to S7.

No discernible variations were noted when subgroups were assessed by factors such as study design and intervention duration.

### Sensitivity analysis

**Figure 5:**
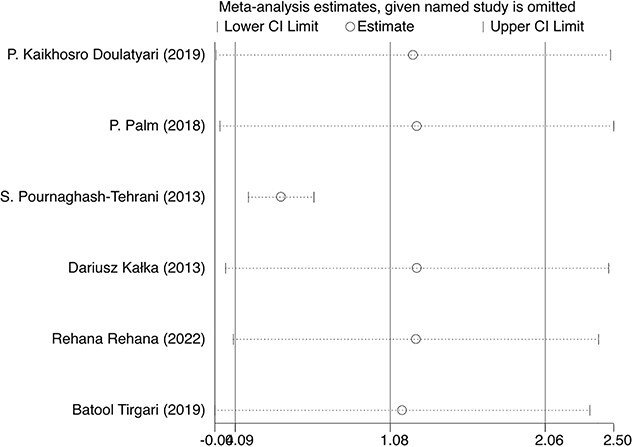
Sensitivity analysis of studies in the meta-analysis: this sensitivity analysis plot illustrates the stability of the combined effect size for the included studies when individual studies are excluded. The plot highlights the consistency of effect sizes within the 95% CI of the overall analysis, except for the notable deviation seen with the exclusion of Pournaghash-Tehrani et al.^20^ The effect size estimate (dppc2) and 95% CIs were calculated via a random effects meta-analysis.

The sensitivity analysis outcomes emphasized that, when any individual study was excluded, the consolidated effect size for the remaining studies remained consistently within the 95% CI of the combined studies’ effect size, underscoring the robustness of our analytical approach. However, an exception was observed upon the exclusion of the Pournaghash-Tehrani et al^20^ study. Despite its pooled effect size still residing within the confidence bounds, the deviation from the combined effect size of all studies was noteworthy. This variation became even more discernible when compared with the results obtained after the exclusion of each of the other singular studies, as depicted in Figure 5.

### Publication bias

The results from Begg’s and Egger’s tests produced *P* values of .06 and .07, respectively. Given that these values are less than the significance level of .1, they suggest a possible risk of publication bias. However, a subsequent trim-and-fill analysis further supported a low risk of publication bias, with no trimming required and the pooled estimate remaining unchanged (*P* < .001). Given that the trim-and-fill method is considered more reliable in assessing publication bias, the overall analysis indicates that our study has a low risk of such bias.

### Quality of evidence

Using the GRADE framework, we assessed the quality of evidence for the ED outcome based on the Morris dppc2 metric. All studies integrated into the meta-analysis were considered, with the exception of Pournaghash-Tehrani et al^20^ due to its high RoB for the selective outcome reporting domain and notable discrepancies observed in the sensitivity analysis results. Ultimately, the quality of evidence was found to be low. For another outcome yielded by a distinct analysis with the IIEF-5 and WMD, the evidence quality was deemed to be very low, as delineated in Table 3.

**Table 3:** GRADE-based evaluation of evidence quality.

		**Downgrading Items**				**No. of patients**		**Measure of effect (95% CI)**		
**No. of studies**	**Design**	**Risk of bias**	**Inconsistency**	**Imprecision**	**Publication bias**	**Cardiac rehabilitation**	**Usual care**	**WMD**	**SMD: dppc2**	**Quality of evidence**
ED: 5^a^	RCT and non-RCT	Very serious	Not serious	Not serious	Unlikely	311	247	—	0.38 (0.17, 0.59)	Low
ED: 3^b^	RCT	Serious	Very serious	Very serious	Unlikely	221	153	5.58 (–1.90, 13.07)	—	Very low

Abbreviations: ED, erectile dysfunction; GRADE, Grading of Recommendations Assessment, Development and Evaluation; IIEF-5, 5-item International Index of Erectile Function; RCT, randomized controlled trial; SMD, standardized mean difference; WMD, weighted mean difference.

a Studies conducted without selective outcome reporting bias.

b Studies used the IIEF-5 to assess erectile dysfunction.

## Discussion

Our systematic review and meta-analysis have demonstrated statistically significant improvements in erectile function among cardiovascular patients undergoing CR, as quantified by the IIEF. This finding is further supported by 2 additional studies that were not in the meta-analysis due to differences in outcome reporting and assessment methods. Despite their exclusion, these studies showed comparable improvements in erectile function, reinforcing the potential benefits of CR. These results highlight the potential of CR programs in addressing ED in this patient population.

CR interventions vary significantly across the studies but generally incorporate structured physical exercise and educational components aimed at improving cardiovascular and sexual health. Begot et al^16^ implemented a home-based walking program, with participants progressively increasing their walking duration over 4 weeks. Belardinelli et al^22^ described a supervised cycle ergometer exercise program, with patients cycling at 60% peak VO_2_ thrice weekly for 60 minutes each session. Kaikhosro Doulatyari et al^21^ combined standard CR with Kegel exercises and stress-coping strategies. Pournaghash-Tehrani et al^20^ utilized the PRECEDE-PROCEED model for educational sessions alongside regular CR. Rehana et al^24^ incorporated health education and monitored physical activities. Tirgari et al^23^ focused on education and exercise, including pelvic floor muscle activities postdischarge. Kałka et al^15^ emphasized interval endurance training on a cycle ergometer, and Palm et al^19^ combined physical exercise training with pelvic floor exercises and psychoeducational consultations. These diverse interventions highlight the multifaceted approach of CR programs in addressing cardiovascular and ED outcomes.

CR improves ED and aims to reduce the recurrence of cardiovascular events.^39-41^ This dual benefit is crucial, as ED often coexists with cardiovascular events, indicating underlying vascular disease.^3^^,^^42^ By addressing cardiovascular and sexual health, comprehensive CR programs significantly enhance overall patient outcomes.^19^ In patients with an established diagnosis of CVD, ED is likely a result of the same underlying pathophysiologic processes that cause CVD, such as atherosclerosis and endothelial dysfunction.^3^^,^^5^^,^^7^ Additionally, medications used in the treatment of CVD, such as certain antihypertensives, can contribute to the occurrence or exacerbation of ED.^6^ The importance of ED in CVD is underscored by its impact on men’s self-esteem, sex life, and overall QoL.^43^ Nonpharmacologic interventions for ED have shown promising results. These methods include sex therapy, the use of vacuum erection devices, penile prosthesis implantation, and penile vascular surgery, as well as lifestyle changes such as exercise, weight loss, smoking cessation, and dietary improvements (eg, healthier dietary patterns), all of which have been demonstrated to effectively improve ED.^44-46^

Some recent evidence underlines CR’s effectiveness, a nonpharmacologic intervention, in improving erectile function.^15^^,^^16^^,^^19-24^ Physical activity, as a core component of CR, has been shown to reduce vascular ED, which is a major biopsychosocial problem for men with cardiac diseases, affecting their QoL.^25^ The findings of the present study reinforce the importance of incorporating comprehensive CR programs, particularly sexual rehabilitation, in the treatment of patients with CVD to address not only cardiovascular health but also ED.

This study aligns with prior research, such as the studies by Park et al^29^ and Boothby et al,^27^ in demonstrating the positive impact of cardiac or sexual rehabilitation on sexual dysfunction in patients with CVD.^27^^,^^29^ Our study, focusing on CR programs, offers a nuanced perspective as compared with Park’s broader range of interventions and Boothby’s narrative review limited by heterogeneity. While the Park et al meta-regression analysis did not identify significant influencing variables, our study employed subgroup analyses and used the GRADE system for evaluating evidence quality.

An analysis on studies using the IIEF-5 with the effect size of WMD revealed a 6-score improvement in erectile function. While not statistically significant, it exceeds the minimal clinically significant difference of 4 defined for IIEF by Rosen et al.^47^ Considering that the IIEF-5 is about 5/6 the size of the IIEF, this effect size indicates clinical significance. However, this result is limited by several methodological issues: the small number of studies, significant heterogeneity, and a wide 95% CI (–1.90 to 13.07). These limitations highlight the need for caution in interpreting the results. The significant heterogeneity and wide confidence interval suggest the possible clinical insignificance of CR and, in some cases, not only improving but potentially worsening ED by 2 scores. Consequently, the evidence quality for this outcome per the GRADE approach is very low and inconclusive.

We identified disparities based on the country of study origin. Developing countries such as Iran and Indonesia, identified as lower-middle-income economies by their gross national income,^48^ showed a pronounced effect size. In contrast, studies from Denmark and Poland, high-income economies, indicated less significant effects. Several reasons might explain these differences. High-income, with extensive health care investments, offers better health care infrastructures as compared with lower-middle-income countries.^49-54^ In low-income regions, patients often present with advanced illnesses, and limited health care access might create a larger margin for CR improvement,^55-57^ rationalizing the pronounced effect sizes observed. Additionally, patient perceptions and sociocultural factors, more than clinical advice, influence decisions to participate in CR programs.^58^^,^^59^ In developing countries, cultural preferences for alternative remedies can enhance the effectiveness of these programs.^60^^,^^61^ Addressing these sociocultural contexts is essential for optimizing CR outcomes.^62^

Studies employing the IIEF-5 for assessing erectile function displayed a larger effect size than those using IIEF, likely because IIEF-5, being a more concise yet comprehensive 5-item measure, allows for a more nuanced evaluation of erectile function.^63-66^ Additionally, a dose-response analysis showed a negative correlation between the number of CR sessions and efficacy in improving erectile function. Initial sessions seemed to have a more pronounced effect, potentially due to higher motivation or a placebo effect, while prolonged sessions might lead to fatigue, hormonal shifts, or a ceiling effect, suggesting a need for further research to optimize CR duration and intensity for erectile function improvement.^67-71^

Domain 5 (selective outcome reporting bias) of the Cochrane Collaboration tool in our RoB analysis highlighted that all but 1 study (Pournaghash-Tehrani et al^20^) had low RoB. Excluding this study significantly affected the overall measure, decreasing heterogeneity with the *I*^2^ statistic dropped from 95% to 0%, implying selective outcome reporting bias as the source of heterogeneity. This emphasized the importance of a priori approaches in research design, where methodologies and analyses are predefined, to avoid biases and maintain consistency and transparency.

As for the quality of evidence assessed through the GRADE framework, it was determined based on the remaining 5 studies. Intriguingly, after this exclusion, the recalibrated dppc2 effect size 95% CI was found to fall within a low effect interpretational range. We therefore conclude that CR has a statistically significant positive impact on ED in patients with cardiovascular conditions, although the effect size is considered low. It is worth noting, however, that the overall quality of our primary evidence remains low, according to GRADE criteria. This is primarily due to the high RoB observed in all but 1 study (Palm et al^19^).

A key strength of our study is its alignment and support for the recommendations of the latest guidelines by the joint committee of the American Heart Association and the American College of Cardiology on the treatment of patients with chronic coronary disease,^72^ including a class 2a recommendation (moderate strength), supported by level B-NR quality of evidence from nonrandomized studies. Specifically, the guidelines suggest CR with sexual counseling for men with chronic coronary disease and emphasize that a sexual rehabilitation program is linked to enhanced sexual function according to the IIEF. Our present study not only reinforces this existing recommendation but also substantially enhances its evidential foundation, utilizing a rigorous meta-analytic methodology governed by the GRADE framework. Another strength of our study is the identification of selective outcome reporting bias as a major source of heterogeneity in our subgroup analysis.

Despite the encouraging outcomes, several limitations must be acknowledged. First, the quality of the studies was generally low, marked by a high RoB in multiple domains. Additionally, small sample sizes and substantial heterogeneity—partly due to inconsistent outcome measures, such as the IIEF, IIEF-5, and researcher-crafted questionnaires—complicate the generalizability of our findings. Importantly, economic and cultural considerations were confined to contrasts between developed and developing nations, with a complete absence of data from underdeveloped countries.

Our review highlights the lack of standardization in the samples across studies, with varying definitions and measurements of ED. Better characterization of the population that benefits most from CR is essential. Future studies should aim to standardize protocols and include diverse patient populations to enhance generalizability.

Future research should focus on rigorous RCTs incorporating sociocultural and economic factors, using consistent measures such as the IIEF-5. Additionally, investigating the dose-response relationship in CR programs is crucial for determining the most effective intervention duration. Importantly, studies must rigorously address selective outcome reporting bias by adhering to predefined protocols and a priori approach, ensuring the integrity and reliability of findings.

## Conclusion

This study offers pivotal and conclusive evidence supporting the efficacy of CR in improving ED among individuals with cardiovascular conditions. While the observed effects were statistically significant, they were modest in magnitude, necessitating a nuanced interpretation. Our findings implied that the higher number of sessions does not improve the CR effect on the ED. These findings reinforce existing guidelines and may notably affect clinical practice, advocating for the inclusion of CR in managing cardiovascular conditions.

## Supplementary Material

Bostan_Heidari-Supplementary_Figures_and_Method_qfae043

## Data Availability

The data supporting this study’s findings are available from the corresponding author upon reasonable request.
