# Supplementary material for: Impact of cardiac rehabilitation on erectile dysfunction in cardiovascular patients: a systematic review and meta-analysis
Source: Sex Med. 2024 Jul 1;12(3):qfae043. doi: 10.1093/sexmed/qfae043 (PMC11215551; doi:10.1093/sexmed/qfae043)
Supplement: Bostan_Heidari-Supplementary_Figures_and_Method_qfae043 [file bostan_heidari-supplementary_figures_and_method_qfae043.docx]

**Supplementary Figures and Methods**

**Table of Contents**

**Supplementary Figure 1,** dppc2-Based Forest Plot Assessing CR effects on ED ……………………………………..………….………..……………. **Page 1**

**Supplementary Figure 2,** Stratified Forest Plot Assessing the dppc2 Impact of CR on ED Based on Studies’ Overall RoB ………….………….… **Page 2**

**Supplementary Figure 3,** Forest Plot of Sequence Generation Domain Assessment of RoB by dppc2 Metric …………………...……..…………… **Page 3**

**Supplementary Figure 4,** Forest Plot of Allocation Concealment Domain Assessment of RoB by dppc2 Metric …………………………………..… **Page 4**

**Supplementary Figure 5,** Forest Plot of Blinding of Outcome Assessment Domain Evaluation of RoB by dppc2 Metric …………………………… **Page 5**

**Supplementary Figure 6,** Forest Plot of Incomplete Outcome Data Domain Assessment of RoB by dppc2 Metric ………………………..……...… **Page 6**

**Supplementary Figure 7,** Forest Plot of Similarity of Groups at Baseline Domain Assessment of RoB by dppc2 Metric …………………...…….… **Page 7**

**Supplementary Method 1,** PRISMA 2020 for Abstracts Checklist ……………………………………………………………….……………….,,,,,,,,,,… **Page 8**

**Supplementary Method 2,** PRISMA 2020 Checklist ……………………………………………………………………………………….…….……….… **Page 10**

**Supplementary Method 3,** PRISMA-S Checklist ………………………………………………………………………………...………………….…….… **Page 17**

**Supplementary Method 4,** Database-Specific Search Syntaxes and Corresponding Result Counts …………………………….……………...….… **Page 20**

**Supplementary Figure 1** dppc2-Baed Forest Plot Assessing Cardiac Rehabilitation effects on Erectile Dysfunction

**Caption** This forest plot summarizes the cardiac rehabilitation effect on erectile dysfunction, quantified by dppc2 in the included studies. It shows individual study estimates with their 95% confidence intervals and the aggregate effect size derived from a random-effects model. The I^2^ statistic denotes interstudy heterogeneity. Study weights reflect the random-effects model computations. Abbreviation: CI, Confidence Interval; ES, Effect Size.

**Supplementary Figure 2** Stratified Forest Plot Assessing the dppc2 Impact of Cardiac Rehabilitation on Erectile Dysfunction Based on Studies’ Overall Risk of Bias Assessment

**Caption** Forest plot displaying the effect of cardiac rehabilitation on erectile dysfunction, stratified by the overall risk of bias assessment of the included studies and measured by the dppc2 metric. Abbreviations: CI, Confidence Interval; ES, Effect Size.

**Supplementary Figure 3** Forest Plot of Sequence Generation Domain Assessment of Risk of Bias by dppc2 Metric

**Caption** This figure depicts the forest plot of the “Sequence Generation” domain (D1) from the Cochrane risk of bias tool across studies included in the meta-analysis. The plot stratifies studies by their methodological approach to this domain, assessing its impact on the dppc2 metric. Abbreviations: CI, Confidence Interval; ES, Effect Size.

**Supplementary Figure 4** Forest Plot of Allocation Concealment Domain Assessment of Risk of Bias by dppc2 Metric

**Caption** This figure depicts the forest plot of the “Allocation Concealment” domain (D2) from the Cochrane risk of bias tool across studies included in the meta-analysis. The plot stratifies studies by their methodological approach to this domain, assessing its impact on the dppc2 metric. Abbreviations: CI, Confidence Interval; ES, Effect Size.

**Supplementary Figure 5** Forest Plot of Blinding of Outcome Assessment Domain Evaluation of Risk of Bias by dppc2 Metric

**Caption** This figure depicts the forest plot of the “Blinding of Outcome Assessment” domain (D3) from the Cochrane risk of bias tool across studies included in the meta-analysis. The plot stratifies studies by their methodological approach to this domain, assessing its impact on the dppc2 metric. Abbreviations: CI, Confidence Interval; ES, Effect Size.

**Supplementary Figure 6** Forest Plot of Incomplete Outcome Data Domain Assessment of Risk of Bias by dppc2 Metric

**Caption** This figure depicts the forest plot of the “Incomplete Outcome Data” domain (D4) from the Cochrane risk of bias tool across studies included in the meta-analysis. The plot stratifies studies by their methodological approach to this domain, assessing its impact on the dppc2 metric. Abbreviations: CI, Confidence Interval; ES, Effect Size.

**Supplementary Figure 7** Forest Plot of Similarity of Groups at Baseline Domain Assessment of Risk of Bias by dppc2 Metric

**Caption** This figure depicts the forest plot of the “Similarity of Groups at Baseline” domain (D6) from the Cochrane risk of bias tool across studies included in the meta-analysis. The plot stratifies studies by their methodological approach to this domain, assessing its impact on the dppc2 metric. Abbreviations: CI, Confidence Interval; ES, Effect Size.

**Supplementary Method 1,** PRISMA 2020 for Abstracts Checklist

| **Section and Topic** | **Item #** | **Checklist item** | **Location reported** |
| --- | --- | --- | --- |
| **TITLE** | | |  |
| Title | 1 | Identify the report as a systematic review. | Line 25 |
| **BACKGROUND** | | |  |
| Objectives | 2 | Provide an explicit statement of the main objective(s) or question(s) the review addresses. | Lines 27-29 |
| **METHODS** | | |  |
| Eligibility criteria | 3 | Specify the inclusion and exclusion criteria for the review. | Lines 35, 36 |
| Information sources | 4 | Specify the information sources (e.g. databases, registers) used to identify studies and the date when each was last searched. | Lines 31, 32 |
| Risk of bias | 5 | Specify the methods used to assess risk of bias in the included studies. | Lines 32, 33 |
| Synthesis of results | 6 | Specify the methods used to present and synthesise results. | Lines 33, 34 |
| **RESULTS** | | |  |
| Included studies | 7 | Give the total number of included studies and participants and summarise relevant characteristics of studies. | Lines 36 |
| Synthesis of results | 8 | Present results for main outcomes, preferably indicating the number of included studies and participants for each. If meta-analysis was done, report the summary estimate and confidence/credible interval. If comparing groups, indicate the direction of the effect (i.e. which group is favoured). | Lines 38-42 |
| **DISCUSSION** | | |  |
| Limitations of evidence | 9 | Provide a brief summary of the limitations of the evidence included in the review (e.g. study risk of bias, inconsistency and imprecision). | Lines 42-44 |
| Interpretation | 10 | Provide a general interpretation of the results and important implications. | Lines 44-47 |
| **OTHER** | | |  |
| Funding | 11 | Specify the primary source of funding for the review. | N/A |
| Registration | 12 | Provide the register name and registration number. | Line 50 |

**Supplementary Method 2,** PRISMA 2020 Checklist

| **Section and Topic** | **Item #** | **Checklist item** | **Location where item is reported** |
| --- | --- | --- | --- |
| **TITLE** | | |  |
| Title | 1 | Identify the report as a systematic review. | Line 25 |
| **ABSTRACT** | | |  |
| Abstract | 2 | See the PRISMA 2020 for Abstracts checklist. | PRISMA 2020 for Abstracts checklist file |
| **INTRODUCTION** | | |  |
| Rationale | 3 | Describe the rationale for the review in the context of existing knowledge. | Lines 79-116 |
| Objectives | 4 | Provide an explicit statement of the objective(s) or question(s) the review addresses. | Lines 117-119 |
| **METHODS** | | |  |
| Eligibility criteria | 5 | Specify the inclusion and exclusion criteria for the review and how studies were grouped for the syntheses. | Lines 167-170 |
| Information sources | 6 | Specify all databases, registers, websites, organisations, reference lists and other sources searched or consulted to identify studies. Specify the date when each source was last searched or consulted. | Lines 134, 135  137-139  148-151 |
| Search strategy | 7 | Present the full search strategies for all databases, registers and websites, including any filters and limits used. | Lines 136, 137 |
| Selection process | 8 | Specify the methods used to decide whether a study met the inclusion criteria of the review, including how many reviewers screened each record and each report retrieved, whether they worked independently, and if applicable, details of automation tools used in the process. | Lines 158-160  163-169 |
| Data collection process | 9 | Specify the methods used to collect data from reports, including how many reviewers collected data from each report, whether they worked independently, any processes for obtaining or confirming data from study investigators, and if applicable, details of automation tools used in the process. | Lines 173-177 |
| Data items | 10a | List and define all outcomes for which data were sought. Specify whether all results that were compatible with each outcome domain in each study were sought (e.g. for all measures, time points, analyses), and if not, the methods used to decide which results to collect. | Lines 181-183 |
|  | 10b | List and define all other variables for which data were sought (e.g. participant and intervention characteristics, funding sources). Describe any assumptions made about any missing or unclear information. | Lines 178-183 |
| Study risk of bias assessment | 11 | Specify the methods used to assess risk of bias in the included studies, including details of the tool(s) used, how many reviewers assessed each study and whether they worked independently, and if applicable, details of automation tools used in the process. | Lines 190, 191 |
| Effect measures | 12 | Specify for each outcome the effect measure(s) (e.g. risk ratio, mean difference) used in the synthesis or presentation of results. | Lines 218-221 |
| Synthesis methods | 13a | Describe the processes used to decide which studies were eligible for each synthesis (e.g. tabulating the study intervention characteristics and comparing against the planned groups for each synthesis (item #5)). | Lines 218-221 |
|  | 13b | Describe any methods required to prepare the data for presentation or synthesis, such as handling of missing summary statistics, or data conversions. | N/A |
|  | 13c | Describe any methods used to tabulate or visually display results of individual studies and syntheses. | Lines 216 |
|  | 13d | Describe any methods used to synthesize results and provide a rationale for the choice(s). If meta-analysis was performed, describe the model(s), method(s) to identify the presence and extent of statistical heterogeneity, and software package(s) used. | Lines 212-216 |
|  | 13e | Describe any methods used to explore possible causes of heterogeneity among study results (e.g. subgroup analysis, meta-regression). | Lines 222-223 |
|  | 13f | Describe any sensitivity analyses conducted to assess robustness of the synthesized results. | Lines 224-225 |
| Reporting bias assessment | 14 | Describe any methods used to assess risk of bias due to missing results in a synthesis (arising from reporting biases). | Lines 190-200 |
| Certainty assessment | 15 | Describe any methods used to assess certainty (or confidence) in the body of evidence for an outcome. | Lines 203-209 |
| **RESULTS** | | |  |
| Study selection | 16a | Describe the results of the search and selection process, from the number of records identified in the search to the number of studies included in the review, ideally using a flow diagram. | Lines 229-233; Figure 1 |
|  | 16b | Cite studies that might appear to meet the inclusion criteria, but which were excluded, and explain why they were excluded. | Lines 233-235; Figure 1 |
| Study characteristics | 17 | Cite each included study and present its characteristics. | Lines 236-246; Table 1 |
| Risk of bias in studies | 18 | Present assessments of risk of bias for each included study. | Lines 262-270; Figure 3 |
| Results of individual studies | 19 | For all outcomes, present, for each study: (a) summary statistics for each group (where appropriate) and (b) an effect estimate and its precision (e.g. confidence/credible interval), ideally using structured tables or plots. | Table 2; Figure 2; Supplementary Figure 1 |
| Results of syntheses | 20a | For each synthesis, briefly summarise the characteristics and risk of bias among contributing studies. | Lines 262-270; Figure 3 |
|  | 20b | Present results of all statistical syntheses conducted. If meta-analysis was done, present for each the summary estimate and its precision (e.g. confidence/credible interval) and measures of statistical heterogeneity. If comparing groups, describe the direction of the effect. | Lines 249-259; Figure 2; Supplementary Figure 1; Table 2 |
|  | 20c | Present results of all investigations of possible causes of heterogeneity among study results. | Lines 273-284, Lines 287-297; Table 2; Figure 4; Supplementary Figure 2-7 |
|  | 20d | Present results of all sensitivity analyses conducted to assess the robustness of the synthesized results. | Lines 300-307; Figure 5 |
| Reporting biases | 21 | Present assessments of risk of bias due to missing results (arising from reporting biases) for each synthesis assessed. | Lines 287-297; Figure 4 |
| Certainty of evidence | 22 | Present assessments of certainty (or confidence) in the body of evidence for each outcome assessed. | Lines 318-324; Table 3 |
| **DISCUSSION** | | |  |
| Discussion | 23a | Provide a general interpretation of the results in the context of other evidence. | Lines 327-331 |
|  | 23b | Discuss any limitations of the evidence included in the review. | Lines 358-362; Lines 408-414 |
|  | 23c | Discuss any limitations of the review processes used. | Lines 408-414 |
|  | 23d | Discuss implications of the results for practice, policy, and future research. | Lines 397-406; Lines 415-419 |
| **OTHER INFORMATION** | | |  |
| Registration and protocol | 24a | Provide registration information for the review, including register name and registration number, or state that the review was not registered. | Lines 125-127 |
|  | 24b | Indicate where the review protocol can be accessed, or state that a protocol was not prepared. | Lines 125-127 |
|  | 24c | Describe and explain any amendments to information provided at registration or in the protocol. | N/A |
| Support | 25 | Describe sources of financial or non-financial support for the review, and the role of the funders or sponsors in the review. | Line 443 |
| Competing interests | 26 | Declare any competing interests of review authors. | Line 446 |
| Availability of data, code and other materials | 27 | Report which of the following are publicly available and where they can be found: template data collection forms; data extracted from included studies; data used for all analyses; analytic code; any other materials used in the review. | Lines 122-123 |

**Supplementary Method 3,** PRISMA-S Checklist

| **Section/topic** | **#** | **Checklist item** | **Location(s) Reported** |
| --- | --- | --- | --- |
| **INFORMATION SOURCES AND METHODS** | | | |
| Database name | 1 | Name each individual database searched, stating the platform for each. | Lines 133-135 |
| Multi-database searching | 2 | If databases were searched simultaneously on a single platform, state the name of the platform, listing all of the databases searched. | N/A |
| Study registries | 3 | List any study registries searched. | Lines 137-140 |
| Online resources and browsing | 4 | Describe any online or print source purposefully searched or browsed (e.g., tables of contents, print conference proceedings, web sites), and how this was done. | Lines 148, 149 |
| Citation searching | 5 | Indicate whether cited references or citing references were examined, and describe any methods used for locating cited/citing references (e.g., browsing reference lists, using a citation index, setting up email alerts for references citing included studies). | Lines 149-151 |
| Contacts | 6 | Indicate whether additional studies or data were sought by contacting authors, experts, manufacturers, or others. | Lines 184-187 |
| Other methods | 7 | Describe any additional information sources or search methods used. | Lines 149-151 |
| **SEARCH STRATEGIES** | | | |
| Full search strategies | 8 | Include the search strategies for each database and information source, copied and pasted exactly as run. | Line 147  Supplementary Method 4 |
| Limits and restrictions | 9 | Specify that no limits were used, or describe any limits or restrictions applied to a search (e.g., date or time period, language, study design) and provide justification for their use. | Lines 136-137 |
| Search filters | 10 | Indicate whether published search filters were used (as originally designed or modified), and if so, cite the filter(s) used. | N/A |
| Prior work | 11 | Indicate when search strategies from other literature reviews were adapted or reused for a substantive part or all of the search, citing the previous review(s). | N/A |
| Updates | 12 | Report the methods used to update the search(es) (e.g., rerunning searches, email alerts). | N/A |
| Dates of searches | 13 | For each search strategy, provide the date when the last search occurred. | Line 135 |
| **PEER REVIEW** | | | |
| Peer review | 14 | Describe any search peer review process. | N/A |
| **MANAGING RECORDS** | | | |
| Total Records | 15 | Document the total number of records identified from each database and other information sources. | Figure 1 |
| Deduplication | 16 | Describe the processes and any software used to deduplicate records from multiple database searches and other information sources. | Lines 154-156 |

**Supplementary Method 4,** Database-Specific Search Syntaxes and Corresponding Result Counts

All database searches were conducted on November 30, 2022.

- **PubMed, 118 Results**

(“Cardiac Rehabilitation*”[all] OR (Cardiac[all] AND Rehabilitation*[all]) OR “Cardiovascular Rehabilitation*”[all] OR (Cardiovascular[all] AND Rehabilitation*[all])) AND (“Erectile Dysfunction”[all] OR (Erectile[all] AND Dysfunction[all]) OR “Male Impotence”[all] OR (Male[all] AND Impotence[all]) OR “Male Sexual Impotence”[all] OR (“Male Sexual”[all] AND Impotence[all]) OR (Male[all] AND “Sexual Impotence”[all]) OR Impotence[all]) AND 2000/01/01:2022/11/30[dp]

- **Scopus, 1685 Results**

(TITLE-ABS(“Cardiac Rehabilitation*”) OR (ALL(Cardiac) AND ALL(Rehabilitation*)) OR ALL(“Cardiovascular Rehabilitation*”) OR (TITLE-ABS(Cardiovascular) AND ALL(Rehabilitation*))) AND (TITLE-ABS(“Erectile Dysfunction”) OR (ALL(Erectile) AND ALL(Dysfunction)) OR ALL(“Male Impotence”) OR (ALL(Male) AND ALL(Impotence)) OR ALL(“Male Sexual Impotence”) OR (ALL(“Male Sexual”) AND ALL(Impotence)) OR (ALL(Male) AND ALL(“Sexual Impotence”)) OR TITLE-ABS(Impotence)) AND PUBYEAR > 1999 AND PUBYEAR < 2023 AND NOT PUBDATETXT(“November 2022” OR “December 2022”)

- **Embase, 268 Results**

(‘Cardiac Rehabilitation*’ OR (Cardiac AND Rehabilitation*) OR ‘Cardiovascular Rehabilitation*’ OR (Cardiovascular AND Rehabilitation*)) AND (‘Erectile Dysfunction’ OR (Erectile AND Dysfunction) OR ‘Male Impotence’ OR (Male AND Impotence) OR ‘Male Sexual Impotence’ OR (‘Male Sexual’ AND Impotence) OR (Male AND ‘Sexual Impotence’) OR Impotence) AND [2000-2022]/py

- **Web of Science, 108 Results**

(ALL=(“Cardiac Rehabilitation*”) OR (ALL=(Cardiac) AND ALL=(Rehabilitation*)) OR ALL=(“Cardiovascular Rehabilitation*”) OR (ALL=(Cardiovascular) AND ALL=(Rehabilitation*))) AND (ALL=(“Erectile Dysfunction”) OR (ALL=(Erectile) AND ALL=(Dysfunction)) OR ALL=(“Male Impotence”) OR (ALL=(Male) AND ALL=(Impotence)) OR ALL=(“Male Sexual Impotence”) OR (ALL=(“Male Sexual”) AND ALL=(Impotence)) OR (ALL=(Male) AND ALL=(“Sexual Impotence”)) OR ALL=(Impotence)) AND PY=(2000-2022)

- **Cochrane Central Register of Controlled Trials (CENTRAL), 26 Results**

(“Cardiac Rehabilitation*” OR (Cardiac AND Rehabilitation*) OR “Cardiovascular Rehabilitation*” OR (Cardiovascular AND Rehabilitation*)) AND (“Erectile Dysfunction” OR (Erectile AND Dysfunction) OR “Male Impotence” OR (Male AND Impotence) OR “Male Sexual Impotence” OR (“Male Sexual” AND Impotence) OR (Male AND “Sexual Impotence”) OR Impotence)

- **ProQuest, 33 Results**

(AB,TI(“Cardiac Rehabilitation*”) OR (ALL,FT(Cardiac) AND ALL,FT(Rehabilitation*)) OR ALL,FT(“Cardiovascular Rehabilitation*”) OR (AB,TI(Cardiovascular) AND ALL,FT(Rehabilitation*))) AND (AB,TI(“Erectile Dysfunction”) OR (ALL,FT(Erectile) AND ALL,FT(Dysfunction)) OR ALL,FT(“Male Impotence”) OR (ALL,FT(Male) AND ALL,FT(Impotence)) OR ALL,FT(“Male Sexual Impotence”) OR (ALL,FT(“Male Sexual”) AND ALL,FT(Impotence)) OR (ALL,FT(Male) AND ALL,FT(“Sexual Impotence”)) OR AB,TI(Impotence)) AND YR(20000101-20221130)

- **ClinicalTrials(dot)gov, 28 Results**

*Condition*

“cardiovascular disease” OR (cardiovascular AND disease*)

*Outcome*

“Erectile Dysfunction” OR (Erectile AND Dysfunction) OR “Male Impotence” OR (Male AND Impotence) OR “Male Sexual Impotence” OR (“Male Sexual” AND Impotence) OR (Male AND “Sexual Impotence”) OR Impotence

- **International Standard Randomised Controlled Trial Number (ISRCTN), 57 Results**

(“Cardiac Rehabilitation*” OR (Cardiac AND Rehabilitation*) OR “Cardiovascular Rehabilitation*” OR (Cardiovascular AND Rehabilitation*)) AND (“Erectile Dysfunction” OR (Erectile AND Dysfunction) OR “Male Impotence” OR (Male AND Impotence) OR “Male Sexual Impotence” OR (“Male Sexual” AND Impotence) OR (Male AND “Sexual Impotence”) OR Impotence)

- **International Clinical Trials Registry Platform (ICTRP), 91 Results**

*Condition*

“Cardiovascular Disease*” OR (disease* AND Cardiovascular)

*Intervention*

“Cardiac Rehabilitation*” OR (Cardiac AND Rehabilitation*) OR “Cardiovascular Rehabilitation*” OR (Cardiovascular AND Rehabilitation*)

- **OpenGrey, 72 Results**

(“Cardiac Rehabilitation*” OR (Cardiac AND Rehabilitation*) OR “Cardiovascular Rehabilitation*” OR (Cardiovascular AND Rehabilitation*)) AND (“Erectile Dysfunction” OR (Erectile AND Dysfunction) OR “Male Impotence” OR (Male AND Impotence) OR “Male Sexual Impotence” OR (“Male Sexual” AND Impotence) OR (Male AND “Sexual Impotence”) OR Impotence)

- **Open Access Theses and Dissertations (OATD), 15 Results**

“Cardiac Rehabilitation” AND “Erectile Dysfunction”

- **Key Journal (European Journal of Preventive Cardiology), 45 Results**

*In Title*

(“Cardiac Rehabilitation*” OR (Cardiac AND Rehabilitation*) OR “Cardiovascular Rehabilitation*” OR (Cardiovascular AND Rehabilitation*))

*In Abstract*

(“Erectile Dysfunction” OR (Erectile AND Dysfunction) OR “Male Impotence” OR (Male AND Impotence) OR “Male Sexual Impotence” OR (“Male Sexual” AND Impotence) OR (Male AND “Sexual Impotence”) OR Impotence)
